# Epilepsy hospitalizations and psychiatric comorbidities: a study protocol for a nationwide inpatient analysis

**DOI:** 10.1192/j.eurpsy.2023.2199

**Published:** 2023-07-19

**Authors:** M. Silva, M. Gonçalves-Pinho, A. Rita Ferreira, M. Seabra, A. Freitas, L. Fernandes

**Affiliations:** 1 Faculty of Medicine, University of Porto, Porto, Portugal; 2 CINTESIS@RISE, Department of Clinical Neurosciences and Mental Health, Faculty of Medicine, University of Porto, Porto; 3 Department of Psychiatry and Mental Health, Centro Hospitalar do Tâmega e Sousa, Penafiel; 4 Neurology Department, Centro Hospitalar Universitário de São João; 5 Neurology and Neurosurgery Unit, Department of Clinical Neurosciences and Mental Health; 6 CINTESIS@RISE, Department of Community Medicine, Information and Health Decision Sciences (MEDCIDS), Faculty of Medicine, University of Porto; 7 Psychiatry Service, Centro Hospitalar Universitário de São João, Porto, Portugal

## Abstract

**Introduction:**

Psychiatric comorbidities are highly frequent in patients with epilepsy and are associated with negative outcomes. These comorbid conditions can lower the seizure threshold, increase the risk of treatment-resistant epilepsy, and reduce function and quality of life. Additionally, patients with epilepsy have an increased risk of premature mortality, including due to suicide. In this context, although hospitalizations are common in patients with epilepsy, little information on healthcare utilization associated with comorbid psychopathology is available.

**Objectives:**

To characterize psychiatric comorbidities among all hospitalizations with a primary diagnosis of epilepsy and to analyze their association with key-hospitalization outcomes, including length of stay, in-hospital mortality, estimated hospital charges, and readmissions.

**Methods:**

An observational retrospective study will be performed using an administrative database that comprises de-identified routinely collected hospitalization data from all Portuguese mainland public hospitals. All episodes of inpatients, discharged between 2008-2015, with a primary diagnosis of epilepsy (ICD-9-CM code 345.X) will be selected. Psychiatric comorbidities as secondary diagnoses will be identified, grouped into broader categories as defined by the Clinical Classifications Software for ICD-9-CM, and computed into binary variables. Descriptive, univariate, and multivariate analyses will be used.

**Results:**

Descriptive and analytical statistics will be conducted to describe and characterize this sample of hospitalizations. Sociodemographic variables such as age at admission, sex, and place of residence will be characterized. Multivariate models will be used to quantify the association between psychiatric comorbidities and hospitalization outcomes, and results will be presented as crude and adjusted odds ratios.

**Conclusions:**

With this nationwide analysis, we expect to better understand the additional burden of psychiatric comorbidities on epilepsy-related hospitalizations, including psychiatric diagnoses that have not been extensively investigated.

**Disclosure of Interest:**

None Declared

